# Removal of Cd(II) from Aquatic System Using *Oscillatoria* sp. Biosorbent

**DOI:** 10.1100/2012/347053

**Published:** 2012-04-30

**Authors:** Seyed Naser Azizi, Abasalt Hosseinzadeh Colagar, Seyede Maryam Hafeziyan

**Affiliations:** ^1^Department of Chemistry, Nano and Biotechnology Research Group, University of Mazandaran, Mazandaran, Babolsar, Iran; ^2^Department of Chemistry, Faculty of Chemistry, University of Mazandaran, Mazandaran, Babolsar, Iran; ^3^Department of Biology, Faculty of Basic Science, Nano and Biotechnology Research Group, University of Mazandaran, Mazandaran, Babolsar, Iran

## Abstract

Biosorption of Cd(II) ions from aqueous solutions by native and dried *Oscillatoria* sp. Cyanobacterium biomass was investigated in the batch mode. The *Oscillatoria* sp. was prepared from Molecular and Cell Laboratory of University of Mazandaran and grown in BG-11 medium. A comparison of Cd(II) adsorption properties of dried with native *Oscillatoria* sp. biomass was made, the dried one showed a higher biosorption capacity and faster kinetic. The influence of solution pH, contact time, biomass concentration, initial metal ion concentration, and presence of coions using dried *Oscillatoria* sp. biomass as well as pretreatment on the biosorption capacity of the biomass were studied. Various pretreatments of *Oscillatoria* sp. increased biosorption of Cd(II) at pH 7 in comparison with native biomass. However, heating at 100°C in a water bath showed significant improvement in Cd(II) biosorption capacity. The experimental biosorption data was well fitted to the Freundlich model compared to the Langmuir model, and the amount of Cd(II) removed from solution increased with increasing Cd(II) concentration. In addition, the dried biomass was investigated for Cd(II) removal from the simulated real sample containing about 14 mg/l Cd(II) at pH 7, under the same experimental condition.

## 1. Introduction

Heavy metal pollution in the aquatic system is one of the most important environmental problems today. They impose serious health risks via their accumulation in living tissues throughout the food chain. The majority of toxic metal pollutants are waste products of industrial and metallurgical processes. [1]. Cadmium is one of the most toxic elements with reported carcinogenic effects in humans. Sources of Cd include wastes from Cd-based batteries, incinerators, and runoff from agricultural soils where phosphate fertilizers are used, since Cd is a common impurity in phosphate fertilizers [2]. It accumulates mainly in the kidney and liver and high concentrations have been found to lead to chronic kidney dysfunction. It induces cell injury and death by interfering with calcium (Ca) regulation in biological systems. It has been found to be toxic to fish and other aquatic organisms. The phenomenon of removal of metal or metalloid species, compounds, and particulates from solution by biological material has been defined as Biosorption [3]. Biosorption as a bioremediation process has received increasing attention in recent years. Biosorption using the biomass of microorganisms is an effective and economic technology for the removal and recovery of Cd(II) and other heavy metal ions from wastewaters. Various biomaterials such as bacteria, fungi, yeasts, and plants have been employed to investigate effective biosorption systems [3, 4].

Biosorption is a process by which living and nonliving microbial cells as well as cellular products such as polysaccharides can be used for heavy metal ions removal from aqueous solutions [3, 5]. The dried, nonliving, or pretreated microbial biomass frequently displays a higher affinity for metal ions compared with living one and seems to be a preferred alternative to the use of living cells in industrial applications for the removal of heavy metal ions from wastewaters. Living cells are likely to be more sensitive to metal ion concentration and adverse operating conditions of pH and temperature. The extent of metal binding is dependent on metal chemistry, nature of binding and metal affinity for binding sites on the cell surface [6]. Furthermore, a constant nutrient supply is required for using living cells. Recovery of metals and regeneration of biosorbent is complicated for living cells. Higher affinity of nonliving cells for metal ions compared with living one probably due to absence of competing protons produced during metabolism [5, 7].

Cyanobacteria are the largest and most diverse group of photosynthetic prokaryotes whose habitats vary from fresh and marine water to terrestrial environments [8]. The microorganism selected for this study is a filamentous cyanobacterium (*Oscillatoria* sp.) that can be used in eliminating heavy metal ions present in waste solutions. The aim of this study was to investigate the effect of experimental parameters such as pH, contact time, temperature, concentration of algal biomass, initial metal concentration, and the various pretreatments of the biomass as well as presence of coions on Cd(II) biosorption capacity of the *Oscillatoria *sp. have been investigated in the batch system.

## 2. Experimental

### 2.1. Microorganism

The *Oscillatoria *sp. culture used in this study was collected from Mazandaran freshwater Rivers and previously isolated in Molecular and Cell laboratory of University of Mazandaran, Iran. Pure culture was grown in BG-11 medium containing NaNO_3 _(1.5 g/L), K_2_HPO_4_ (0.04 g/L), MgSO_4_·7H_2_O (0.075 g/L), CaCl_2_·2H_2_O (0.036 g/L), citric acid (0.006 g/L), ferric ammonium citrate (0.006 g/L), EDTANa_2_ (0.001 g/L), Na_2_CO_3_ (0.02 g/L), and trace metal mix 1 mL/L. The composition of trace metal mix is H_3_BO_3_ (2.86 g/L), MnCl_2_·4H_2_O (1.81 g/L), ZnSO_4_·7H_2_O (0.222 g/L), Na_2_MoO_4_·2H_2_O (0.39 g/L), and CuSO_4_·5H_2_O (0.079 g/L), Cu (NO_3_)_2_·6H_2_O (0.0494 g/L). The medium used for growing blue-green algae in flasks contains only trace amounts of metal ions and allows rich growth [8].

The cells were grown at 25°C under cool white fluorescent light intensity in 12 h light-dark cycle, in BG-11 minimal medium at pH 7.1 and were incubated for 10–15 days in an incubator which is suitable for photosynthesis. The biomass was harvested in exponential phase after 15 days of growth by centrifugation at 10,000 rpm for 10 min in centrifuge (Universal 320R, Hettich, German). Then, the biomass was washed thoroughly with double-distilled water to improve the metal binding properties. The washed algae were then air dried and grounded prior to use and was used for metal biosorption experiments afterwards. To determine the dry weight of the biomass, four 50.00 mg samples of wet biomass (native) were dried to a constant weight at approximately 90°C at least 12 h on tare watch glasses [9].

### 2.2. Reagents and Apparatus

All the chemicals used in media preparation were analytically graded (BDH) and the standard solution of Cd(II) (1000 ppm) for atomic absorption measurements was manufactured by Fluka company. The working solutions were prepared by further diluting the stock solution. To study the effect of pH on the Cd(II) sorption, the following buffers (all 10 mM) at the specific pH values: acetic acid buffer, pH 3–5; PIPES (piperazine-1,4-bis(2-ethane-sulfonic acid)), pH 6-7, and HEPES (2-[4-(2-hydroxyethyl)-1-piperazinyl]-ethanesulfonic acid) buffer, pH 7-8 were used [10]. Hydrochloric acid and tetramethylammonium hydroxide were used for adjusting the pH values and blank samples were used as controls. The pH meter (744 digital pH meters, Metrohm, German) was employed for measuring pH values in the aqueous phase. The metal ion concentration in supernatant phase was analyzed by flame atomic absorption spectrophotometer (SpectrAA-10, Varian, USA).

### 2.3. Pretreatment of Biomass

To investigate the effect of various treatments on Cd(II) biosorption, 3 mg of dried biomass of 2-week-old cells were suspended in 3 mL of double distilled water containing NaOH (0.1 N) and HCl (0.1 N) and heated at 100°C in a water bath then incubated for 10 min at room temperature. The cells were collected by centrifugation (12,000 rpm, 3 min) and washed thoroughly by double-distilled water until the pH decreased to less than 8.0.

### 2.4. Batch Procedure

Batch biosorption experiments were carried out in 50 mL flasks containing 25 mL of Cd(II) solution of initial concentration 10 mg/L (pH 3–8) at 25°C and at 125 rpm for the period of contact time using a shaker incubator (NB-205QF, N-Biotech, Korea). Separation of biomass from metal bearing solution was achieved through centrifugation at 10,000 rpm for 10 min at room temperature. The supernatant was appropriately diluted and the remaining Cd(II) content estimated at 228.8 nm wavelength (slit width 0.5 nm). Metal-free and biomass-free blanks were used as controls and for estimating the exact initial concentration of Cd(II) by dilution. All experiments were repeated three times.

The difference between the initial and remaining metal ion concentrations was assumed to be taken up by the biosorbent. The metal uptake capacity in mg/g (*Q*) was calculated from the initial concentration (*C*_*i*_) and the final (remaining) concentration (*C*_*f*_) of the metal according to the following:

(1) $$Q=\frac{V\left(C_{i}-C_{f}\right)}{M},$$

where *V* is the liquid sample volume and *M* is the biomass dry weight (g) [9].

### 2.5. Equilibrium Studies

The effect of various initial concentrations of Cd(II) ions on the uptake of Cd(II) by dried biomass was also studied within the range of 5 mg/L to 200 mg/L Cd^2+^ at 25°C and at 125 rpm for the period of contact time (optimum conditions of all pertinent factors were used) while maintaining biomass concentration at 0.16 mg/L. The mixtures were adjusted to pH 7. Samples of supernatant were then collected by centrifugation and analyzed by AAS.

### 2.6. Effect of Co-Ions on Cadmium Biosorption

Solutions of Cd(II) (10 mg/L) were prepared individually with one additional metal ion included Ni(II), Co(II), Cu(II), Zn(II) and total of co-ions in an equimolar concentration. Dried biomass (3 mg) was allowed to contact the Cd(II)/co-ion solutions at 25°C and pH 7 for a predetermined duration. Supernatants were collected by centrifugation and analyzed by AAS.

### 2.7. Simulation of a Real Sample Containing Cadmium Ions by River Water

Quantity of measured Cd(II) in river water sample was almost insignificant according to detection limit of the method (LOD = 0.011), without any pretreatments. So, due to simulation of real sample (river water) containing Cd(II) ions, the required amount of Cd(II) was added to river water, manually, and Cd(II) biosorption capacity of dried *Oscillatoria* sp. biomass was determined after preparing.

## 3. Results and Discussion

### 3.1. Effect of pH on the Cadmium Biosorption

The pH of the solution can play a key role in the biosorption of Cd(II) by the cyanobacterium as it influence both metal binding sites on the cell surface and the chemistry of metal in solution [8, 11]. In order to demonstrate the effect of pH on biosorption, the cells equivalent to 3 mg dry wt. of native and dried biomass were placed in contact with 25.0 mL samples of Cd(II) solutions (10 mg/L Cd^2+^) at 25°C and the mixture was adjusted by buffers to a desired pH (3–8). Samples of the supernatant were collected by centrifugation and analyzed by AAS for metal determination.

**Figure 1:**
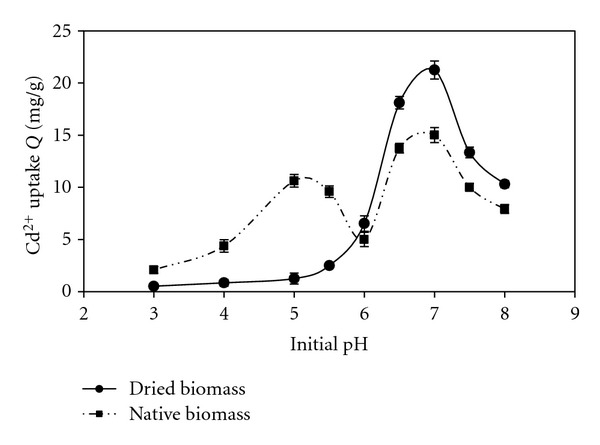
pH profile of Cd(II) uptakes by native and dried biomass at initial concentration of 10 ppm; temperature 25°C.

As shown in Figure 1, cadmium sorption increased with increasing pH in both states and the most cadmium removal occurred at pH 7 for both native and dried biomass. At pH values above 7, the metal ion solubility was lowered due to the formation of metal hydroxides. For native biomass, the Cd(II) uptake increases with increasing of pH up to pH 5 and decreases when pH value was over 5 but increased again with increasing pH within the pH range of 6–7, reaching the highest level at pH 7. The Cd(II) uptake of dried biomass did not increase dramatically up to pH 5.5 but increased rapidly within the pH range of 6–7 and maximum uptake was obtained at pH 7. Therefore, all further studies were performed at pH 7 for native and dried biomass samples. In highly acidic conditions, the cell surface sites are closely linked to the H^+^ ions, thereby making these unavailable for other cations. However, with an increase in pH, there is an increase in ligands with negative charges which results in increased binding of cations [12]. Phosphate groups of lipopolysaccharides (LPSs) and phospholipids present in outer membrane begin to deprotonate around pH 7 and also pH 5 is the pKa range of carboxyl groups so this suggests that these species probably have an important role in cadmium uptake by native biomass however for dried biomass, phosphate groups play a main role in Cd(II) uptake [9, 13]. Pretreatment of biomass at high temperature can modify the surface characteristics either by removing or masking the groups (carboxyl groups) or by exposing more metal-binding sites (phosphate groups). pH values between 4 and 8 are widely accepted as an optimum for metal uptake for almost all types of biomass [8].

### 3.2. Effect of Contact Time on the Cadmium Biosorption

**Figure 2:**
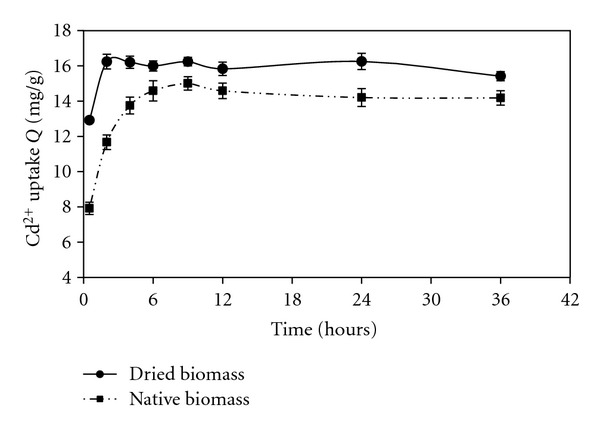
Time-dependency profile of Cd(II) uptakes by native and dried biomass at initial concentration of 10 ppm; temperature 25°C; pH 7.

In order to examine the time-dependent biosorption of Cd(II) by native and dried biomass, samples equivalent to 3 mg dry wt. were placed in contact with 25.0 mL samples of Cd(II) solution (10 mg/L Cd^2+^) adjusted to pH 7 for native and dried biomass. As shown in Figure 2, dried biomass of* Oscillatoria *sp. showed fast kinetic of Cd(II) binding and could adsorb appreciable quantities of Cd(II) during the first 2 h from the aqueous solution with a maximum uptake of 16.24 mg Cd^2+^ per g of dry biomass and after that period, there were no significant changes in the metal uptake, whereas for native biomass the equilibrium was attained after approximately 6 h with uptake value of 14.58 mg Cd^2+^ per g of dry biomass. Equilibrium time is a function of many factors, such as type (number and kind of biosorption sites), size and form of biomass, its physiological state (active or inactive, free or immobilized), and the metal involved in the biosorption system [14].

Cd(II) biosorption could be divided into two stages in both dried and native biomasses: a fast initial rate was followed by a much slower biosorption rate. The fast initial metal biosorption rate was attributed to the surface binding between the negatively charged cell surface ligands and metal cations and the following slower sorption was attributed to the interior penetration. These results are in agreement with the studies of other researchers that reported the uptake was characterized into two stages: a first stage with a high rate and a much slower second one [15–17]. All subsequent uptake experiments were allowed 2 h contact time using dried *Oscillatoria *sp. biomass. Also, since the dried biomass could sequester cadmium to more extent, the absence of an active mechanism dependent on metabolism was suggested so that the main removal was due to passive physico-chemical biosorption. The use of nonliving microbial cells in industrial application may offer some advantages over living cells, such as lower sensitivity to toxic metal ions concentrations and adverse operating conditions [15].

### 3.3. Effect of Temperature on the Cadmium Biosorption

**Figure 3:**
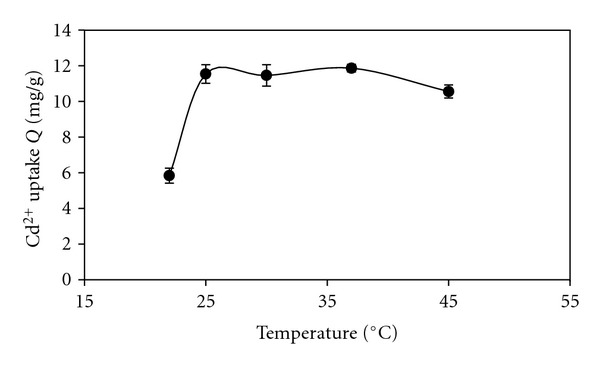
Temprature-dependency profile of Cd(II) uptakes by dried biomass at initial concentration of 10 ppm; pH 7.

The temperature of the adsorption medium could be important for energy-dependent mechanisms in metal biosorption by microbial cells [15]. As shown in Figure 3, sorption of Cd(II) increased with increase in temperature from 22 to 25°C, and with temperature levels ranging from 25 up to 37°C, there were no significant changes in the metal uptake but when temperature was over 37°C, the removal level of Cd(II) reduced. Physical-chemical adsorption reactions were normally exothermic, thus the extent of adsorption generally decreased with further increasing temperature.

### 3.4. Effect of the Biomass Pretreatment on the Cadmium Biosorption

Pretreatment of native biomass using drying at 90°C for 12 h, NaOH (0.1 N), HCl (0.1 N), and heating at 100°C in a water bath for 10 min resulted in an improvement in Cd(II) biosorption in comparison with native biomass.  Table 1 shows the effect of various pretreatments of biomass of* Oscillatoria *sp. on biosorption of Cd(II). It was observed that Q values obtained for all the pretreated biomasses were high in comparison with native biomass. Of the various treatments, heating at 100°C in a water bath for 10 min showed the maximum improvement on Cd(II) sorption. But because dried biomass could be stored for a longer time, all further studies were performed for dried biomass samples. Removal of surface impurities, rupture of cell membrane, and exposure of available binding sites for metal bioadsorption after pretreatment may be the reason for the increase in metal biosorption [5, 18, 19]. Also degraded cells would offer a larger available surface area and expose the intracellular components to more surface binding sites because of the destruction of the cell membranes [3, 20].

### 3.5. Effect of the Biomass Value on the Cadmium Biosorption

**Figure 4:**
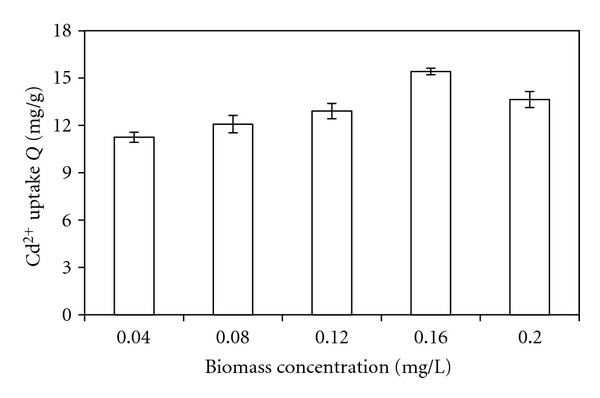
Biomass concentration-dependency profile of Cd(II) uptakes by dried biomass at initial concentration of 10 ppm; temperature 25°C; pH 7.

Figure 4 shows the effect of varying biomass concentration in the biosorption process. Increasing biomass concentration caused an increase in Cd(II) biosorption capacity and the maximum uptake of Cd(II) is obtained at high dose of 0.16 mg/L, but with higher biomass levels, the amount of Cd(II) adsorbed per g of dry biomass decreased. More biomass caused an increase in binding sites, therefore, in the biosorption capacity. However, at higher biomass concentrations aggregates are formed which can reduce the effective biosorption area, as the same result as the references [15, 21], thus less sites are available for metal binding.

### 3.6. Effect of Initial Concentration on the Cadmium Biosorption

**Figure 5:**
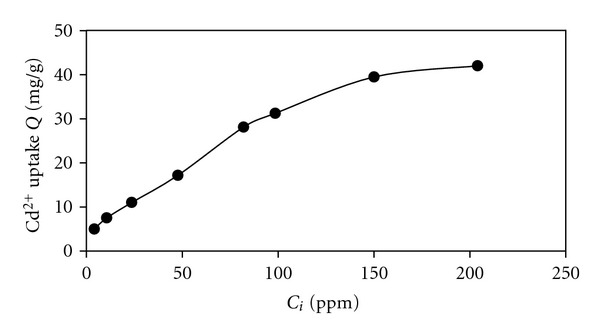
Effect of initial Cd(II) concentration on biosorption of Cd(II) at temperature 25°C; pH 7.

The results showed that the initial Cd(II) concentration remarkably influenced the equilibrium metal uptake. As shown in Figure 5, the maximum retention of Cd(II) by dried biomass, was obtained approximately at Cd(II) concentration of 150 mg/L. The retained Cd(II) increased by increasing the concentration of Cd(II) in solution due to diminishing loading capacity of biomass. At low concentrations, adsorption sites took up the available metal more quickly. However, at higher concentrations, metal ions need to diffuse to the biomass surface by intraparticle diffusion and greatly hydrolyzed ions will diffuse at a slower rate [22].

### 3.7. Langmuir and Freundlich Adsorption Isotherms

The two most commonly used adsorption isotherms are the Langmuir and Freundlich isotherms. The Langmuir isotherm assumes a surface with homogeneous binding sites, equivalent sorption energies, and no interaction between sorbed species. The linear form of Langmuir model is:

(2) $$\frac{1}{Q_{e}}=\frac{1}{Q_{\max}}\left(\frac{1}{bC_{e}}+1\right),$$

where *Q*_*e*_ is the metal uptake at equilibrium, *Q*_max⁡_ the maximum adsorption capacity under the given conditions, *C*_*e*_ the equilibrium concentration, and b relates to the affinity of the sorbate for the binding sites. The Freundlich isotherm is an empirical equation based on an exponential distribution of sorption sites and energies. The linear form of this model takes the form:

(3) $$\log Q_{e}=\log k_{f}+\frac{1}{n}\log C_{e},$$

where *k*_*f*_ and 1/*n* are related to the sorbent capacity and sorption intensity, respectively [23, 24].

**Figure 6:**
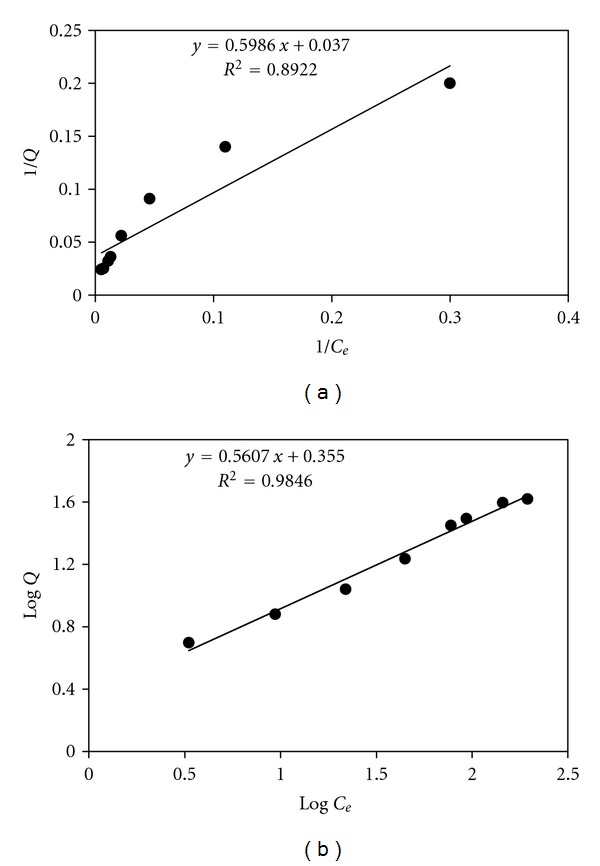
Langmuir (a) and Freundlich (b) adsorption isotherms for *Oscillatoria* sp.

The plots of linearized Langmuir and Freundlich adsorption isotherms were obtained from the conducted equilibrium biosorption experiments at 25°C and pH 7 (as shown in Figure 6). The Langmuir and Freundlich adsorption constants evaluated from these isotherms were given in Table 2. Correlation regression coefficients indicate that the Freundlich isotherm model exhibits better fit to the sorption data of Cd(II) than the Langmuir isotherm model (*R*_2_ = 0.9846). This phenomenon suggests that multilayer sorption takes place on the surface of biomass. The value of *n*, which is related to the distribution of bonded ions on the sorbent surface, represent beneficial adsorption if is between 1 and 10. The *n* value for the biosorbent used was found to be greater than one, indicating that adsorption of Cd(II) is favorable [24].

### 3.8. Effect of Coions on the Cadmium Biosorption

To determine the effect of coions on the biosorption of Cd(II), competitive biosorption experiments were conducted with Ni(II), Co(II), Cu(II), and Zn(II) at equimolar concentrations because of their presence in most industrial effluents [9]. On the other hand, sorption of Cd(II) was influenced by tested cations because they belonged to the same class [3].

**Figure 7:**
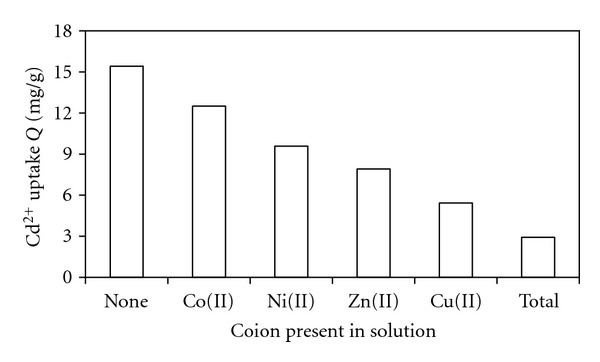
Effect of coions on the uptake of Cd(II) by dried biomass at initial concentration of cadmium 10 ppm; temperature 25°C; pH 7; initial concentration of coions (equimolar): Ni^2+^: 2.9 ppm; Cd^2+^: 5.5 ppm; Cu^2+^: 3.1 ppm; Zn^2+^: 3.3 ppm.

The results presented in Figure 7 indicated that Cd(II) uptake by *Oscillatoria* sp. was reduced in the presence of all the co-ions. Maximum inhibition of Cd(II) ion uptake have been due to the presence of total of co-ions in the solution. The decrease of metal uptake in competitive conditions was thought to be a response to increased competition between same charged species for binding sites of the cells [25].

### 3.9. Determination of Cadmium Biosorption Capacity by Standard Addition Method in the Simulated Real Sample

The real samples were made with initial concentration of about 14 mg/L of Cd(II) ions, adjusted to pH 7, and different amounts of dried biomass were added to solutions and were situated under the condition of the experiment. Metal-free and biomass-free blanks were used as controls and for estimating the exact initial concentration (the exact initial concentration as estimated by atomic absorption measurements, 14.27 mg/L) of Cd(II) by dilution. The standard addition method was used for determination of Cd(II) concentration in the samples. Calibration curves were drawn to determine Cd(II) concentration and also Cd(II) biosorption capacity of *Oscillatoria* sp. biosorbent (data not shown). The Cd(II) biosorption capacity in mg/g (*Q*) was calculated and the results were given in Table 3.

## 4. Conclusions

The goal of this work was to explore the potential use of *Oscillatoria *sp. biomass as a low-cost sorbent for the removal of Cd(II) heavy metal ion from aqueous solutions. Batch experiments showed that *Oscillatoria* sp. has a remarkable ability to take up Cd(II) heavy metal ion. According to the results obtained, biosorption capacities for Cd(II) were strongly dependent on the pH of the solution. The dried biomass of *Oscillatoria* sp. showed a higher biosorption capacity than the native one. Pretreatment of native biomass by different chemical and physical treatment techniques enhanced the biosorption yield. Also, the algal biomass dried at 90°C for 12 h was found to be an efficient biosorbent for removal of Cd(II) ions from aqueous solution. The Cd(II) uptake had a dependence on the initial Cd^2+^ concentration (*C*_*i*_) too. As *C*_*i*_ increased, the saturation point also increased. The biosorption equilibrium data obeyed Freundlich model in the concentration ranges studied. Competitive biosorption studies showed that adsorption yield of Cd(II) ion was reduced by the presence of competing ions in binary metal mixtures. Also, results obtained from this study can surely be used to design a practical and economical process for wastewater treatment. The present paper also emphasizes that cyanobacteria are ideal candidates to be further exploited as being autotrophs, these are easy to cultivate and harvest.

## Figures and Tables

**Table 1:** Effect of biomass pretreatments on biosorption of Cd(II) by* Oscillatoria *sp.

Pretreatment	mg Cd^2+^ biosorbed/g dry biomass
Washing with double-distilled water (native)	11.67 ± 0.43
Drying at 90°C for at least 12 h	13.89 ± 0.36
HCl (0.1 N) treatment for 10 min	13.75 ± 0.64
NaOH (0.1 N) treatment for 10 min	15.00 ± 0.56
Incubation at 100°C in water bath for 10 min	16.67 ± 0.47

**Table 2:** Langmuir and Freundlich parameters for the adsorption isotherms of adsorbent for Cd(II) ion.

Biosorbent	Langmuir			Freundlich		
	*Q*_max⁡_	*b*	*R* ^2^	*k*	*n*	*R* ^2^
Dried biomass of *Oscillatoria *sp.	27.03 ± 7.50	0.062 ± 0.013	0.89	2.26 ± 0.25	1.78 ± 0.09	0.98

**Table 3:** Cd(II) statistical parameters in the simulated real samples for different amounts of dried biomass of *Oscillatoria *sp.

*Oscillatoria* sp. dry wt (mg)	Least square equation	Correlation regression coefficient (*R* ^2^)	Final concentration (mg/L ± *s*_*Cx*_)	Cd(II) biosorption capacity (mg/g)
3	*y* = 0.6004*x* + 0.3244	0.9997	13.51 ± 0.2	6.34 ± 0.26
4	*y* = 0.5816*x* + 0.2996	0.9998	12.88 ± 0.175	8.69 ± 0.3
